# Cytoskeleton-centric protein transportation by exosomes transforms tumor-favorable macrophages

**DOI:** 10.18632/oncotarget.11794

**Published:** 2016-09-01

**Authors:** Zhipeng Chen, Lijuan Yang, Yizhi Cui, Yanlong Zhou, Xingfeng Yin, Jiahui Guo, Gong Zhang, Tong Wang, Qing-Yu He

**Affiliations:** ^1^ Key Laboratory of Functional Protein Research of Guangdong Higher Education Institutes, Institute of Life and Health Engineering, College of Life Science and Technology, Jinan University, Guangzhou 510632, China

**Keywords:** exosomes, tumor-associated macrophages, proteome, transportation, cytoskeleton-centric

## Abstract

The exosome is a key initiator of pre-metastatic niche in numerous cancers, where macrophages serve as primary inducers of tumor microenvironment. However, the proteome that can be exosomally transported from cancer cells to macrophages has not been sufficiently characterized so far. Here, we used colorectal cancer (CRC) exosomes to educate tumor-favorable macrophages. With a SILAC-based mass spectrometry strategy, we successfully traced the proteome transported from CRC exosomes to macrophages. Such a proteome primarily focused on promoting cytoskeleton rearrangement, which was biologically validated with multiple cell lines. We reproduced the exosomal transportation of functional vimentin as a proof-of-concept example. In addition, we found that some CRC exosomes could be recognized by macrophages via Fc receptors. Therefore, we revealed the active and necessary role of exosomes secreted from CRC cells to transform cancer-favorable macrophages, with the cytoskeleton-centric proteins serving as the top functional unit.

## INTRODUCTION

Since the last decade, increasing evidence has suggested that extracellular vesicles (EVs) are critical for the malignant progression of solid tumors, and they represent one of the most competent information deliverers in tumor microenvironment (reviewed in ref. [1–3]). Such information is composed of protein, RNA, DNA, lipids and others, such as small molecules [4, 5]. EVs are now known to be highly heterogenic; for example, Hong *et al* have found that a single cancer cell line can release at least three subtypes of EVs based on deep sequencing analyses [6], which is consistent with their earlier proteomic analyses [7].

As one of the EV subsets, exosomes with the size of 30-150 nm in diameter have been recently found to induce cancer pre-metastatic niche for their integrin-dependent and organ-specific homing behaviors [8]. This niche is also characterized by the extracellular matrix modulation *via* tumor exosomes to facilitate cancer cell motility and invasion [9, 10]. In such a scenario, the exosome works like a “special agent” to light inflammatory and chemotactic signals, preparing for the rendezvous of circulating cancer cells. This expanded the current knowledge of the exosome's role in cell-cell communication between tumor and stroma cells *in situ* [11–16]. Such an exosome-relevant homing feature has now been linked to cancer-associated inflammation at the pre-metastatic site in the lymph node, lung, liver and brain [16–20].

Indeed, other than nucleic acids such as microRNAs, Peinado *et al* have unveiled an exosomally transported oncoprotein of melanoma cells, the receptor tyrosine kinase MET, which initiates long distance inflammation to chemotactically attract circulating cancer cells [21]. Along with numerous other evidence [12, 22–24], the exosome has been recognized to be a specialized group of EVs for the functional transportation of oncoproteins. This moves the field forward as early opinions have deemed the exosome a “garbage can”, merely functioning as a degradation compartment (reviewed in ref. [25, 26]). Although intensively important proteomics profiling studies have been published in the field of cancer exosomes [7, 27–34], what proteins can be ultimately and functionally transported from cancer cells to target cells *via* exosomes has not been investigated in the view of systems biology.

Tumor-associated macrophages (TAMs) *in situ* are known promoters for cancer progression in numerous cancers, including colorectal cancer (CRC) [35–37]. Indeed, acquiring sufficient TAMs should be a critical step for the circulating cancer cells to survive at the homing site. However, the systems mechanism of the exosomally transported proteome from cancer cells to macrophages for developing TAMs is unclear. In this regard, we established an *in vitro* model of CRC exosome-educated mouse bone marrow-derived macrophage (BMM) to acquire cancer-favorable differentiation of BMM. We developed a SILAC-based mass spectrometry (MS) strategy to trace the proteome that was functionally transported from CRC cells to BMMs *via* exosomes. By using multiple cell lines and various biological validations, we depicted the cancer cell-derived exosomal language and the possible mechanism of the exosome recognition by macrophages.

## RESULTS

### CT-26 cell-derived exosomes educate cancer cell-favorable macrophages

**Figure 1:**
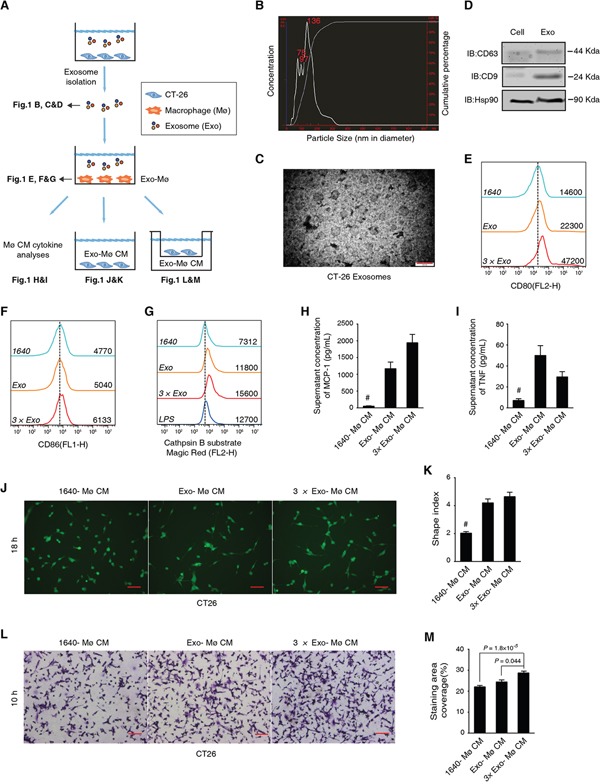
CT-26 cell-derived exosomes are sufficient to transform cancer cell-favorable macrophages **A.** Schematic diagram of the experimental design and results shown in each panel. Mø stands for macrophages, Exo for exosomes, and CM for conditioned media. **B.** Size distribution of CT-26 exosomes determined by NanoSight. **C.** TEM observation of CT-26 exosomes. Scale bar = 200 nm. **D.** Immunoblotting analysis on the exosomal biomarkers CD63, CD9 and Hsp90. **E, F.** Maturation analysis of CT-26 exosome-educated BMMs. Different doses of exosomes, 1× (Exo; the exosome secreted from 3.5×10^6^ cells) or 3× Exo, were added into BMM cultures. FCM results on CD80 (E) and CD86 (F) are respectively shown with fresh RPMI 1640 (1640) cultured cells as the negative control. The mean fluorescent intensities (MFIs) of each group are shown on the right side of each curve. The dashed line indicates the peak value of the negative control group. **G.** FCM assay on cathepsin B activity in live cells. LPS-treated BMMs were used as a positive control. **H, I.** Cytometric bead array assays (CBA) analysis on the BMM cytokine secretion. Statistical results of MCP-1 (H) and TNF (I) are shown. ^#^*P* < 0.01, n = 3, compared with any of the other groups, respectively. **J, K.** Representative CFSE staining image (J) and statistical analysis on the shape index of CT-26 cells (K). Scale bar = 20 μm; ^#^*P* < 0.01, cell n = 80, compared with any of the other groups. **L, M.** Representative images (L) and statistical comparisons of the transmembrane cell staining area of CT-26 cells. Scale bar = 20 μm (M). CT-26 cells were induced to migrate toward different CM. Data are shown as mean ± s.e.m. and n = 3.

With analyses on multiple compartments as illustrated in Figure 1A, we validated a model of mouse CT-26 cell-derived exosomes (CT-26 exosomes) educated macrophages. First, we determined that ∼70% CT-26 exosomes had the size ranging from 30 to 150 nm in diameter (Figure 1B). The maximum size (∼300 nm) was similar to an exosome doublet (150 nm in diameter for each singlet) (Figure 1B). It has been found that NanoSight technology may tend to over-estimate the particle size [38]. With transmission electron microscopy (TEM), we visually confirmed that our CT-26 exosomes were largely with the expected size of 30-150 nm in diameter (Figure 1C). In addition, we confirmed the expression of known exosomal biomarkers of CD63, CD9 and Hsp90 (Figure 1D). Mouse bone marrow cells were allowed to differentiate for 3 days, followed by the addition of CT-26 exosomes and additional 3 days' culture to model the CRC cell exosome-educated macrophages (CEEMs). We observed that CEEMs were characterized by the up-regulation of macrophage maturation biomarkers of CD80 (Figure 1E) and CD86 (Figure 1F), as well as the increase of cathepsin B activity (Figure 1G) as compared with BMMs. Such activation was able to be amplified by adding more CT-26 exosomes (Figure 1E-1G).

Through Cytometric Bead Array (CBA) cytokine array analyses, we found that CEEMs secreted significantly more MCP-1 (Figure 1H; >10 folds) and TNF (Figure 1I) than BMMs, while no statistical difference was observed regarding IL-6, IL-10, IFN-γ, and IL-12p70 (Supplemental Figure S1A). Similar to TNF, MCP-1 is known to promote CRC cell growth [37]. Such intensive MCP-1 secretion led us to posit that the aberrant CEEM activation was favorable to CRC cell migration, which was supported by the analysis of reciprocal effects of CEEM conditioned media (CM) on CT-26 cells (Figure 1J-1M). In general, the CM acquired from CEEM cultures could significantly increase the shape index (Figure 1J&1K), and significantly promote the transmembrane migration of CT-26 cells (Figure 1L&1M). We further found that the exosome could significantly promote the NFκB expression with NFκB-luciferase reporter gene incorporated RAW264.7 cells (Supplemental Figure S1B). Hence, the CT-26 exosome is a sufficient factor to induce tumor-associated inflammation with the aberrant macrophage activation.

### Characterization of exosomally transported proteome

**Figure 2:**
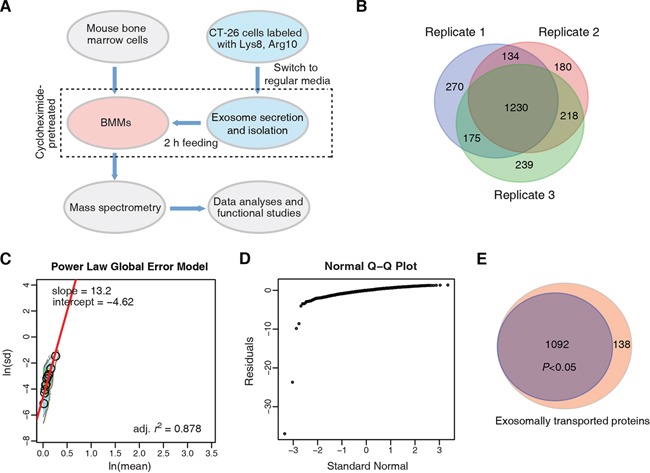
Endpoint tracing of CT-26 exosome transported proteome in macrophages **A.** Strategy for the exosomally transported proteome study. **B.** Venn diagram comparison of exosomally transported proteins identified and quantified in macrophages, acquired from three independent biological replicates. **C.** PLGEM analysis for detecting transported proteins with statistically consistent relative abundance. The protein standard deviations (In(sd)) and means (In(mean)) were linearly fitted by PLGEM. **D.** Q-Q plot. **E.** Venn diagram that shows the fraction of DEPs.

We used the strategy shown in Figure 2A to trace the exosomally transported proteins from CT-26 cells to BMMs. We emphasized that BMMs were pretreated with cycloheximide (CHX) to avoid the reuse of labeled amino acids from degraded peptides (Figure 2A); no significant CHX-induced cytotoxicity in BMMs was observed (Supplemental Figure S2A).

With MS analyses, we identified 4143, 4233 and 4241 proteins (peptide and protein level FDR < 1%, with at least 2 exclusively unique peptides) in three independent experiments, respectively (Supplemental Figure S2B and Supplemental Table S1). Among them, 1809, 1762 and 1862 had quantification information in the same three biological replicates, respectively (Figure 2B); each quantified protein was based on the H/L ratios acquired from at least 2 quantified exclusively unique peptides. There were 1230 such quantified proteins that were consistently identified across the three MS analyses (Supplemental Table S2). We then used Power Law Global Error Model (PLGEM) algorithm [39, 40] to test whether a protein could be transported from CT-26 cells to BMMs *via* the exosome with statistically consistent relative abundance. We found that ln(mean) and ln(SD) of protein H/L ratios had strong linear correlation fitted by the PLGEM, with *r*^2^ = 0.878 (Figure 2C). The H/L ratio residuals showed normal distribution (Supplemental Figure S2C), and the deviation of residuals was independent from the protein relative abundance (Supplemental Figure S2D). Q-Q plot showed that the residuals of the quantified proteins obeyed normal distribution (Figure 2D). These results indicated that PLGEM worked properly to model the above MS quantification data to find differentially expressed proteins (DEPs). Here, we define the DEPs as the heavy proteins identified in BMMs with H/L values, which represent the relative amount of proteins transported from CT-26 cells to macrophages. Accordingly, 1092 out of the 1230 consistently quantified proteins were accepted as significant DEPs by the signal to noise (STN) analysis of PLGEM (*P* < 0.05) (Figure 2E and Supplemental Table S3).

### Pathway analysis of the endpoint exosomally transported proteome

**Figure 3:**
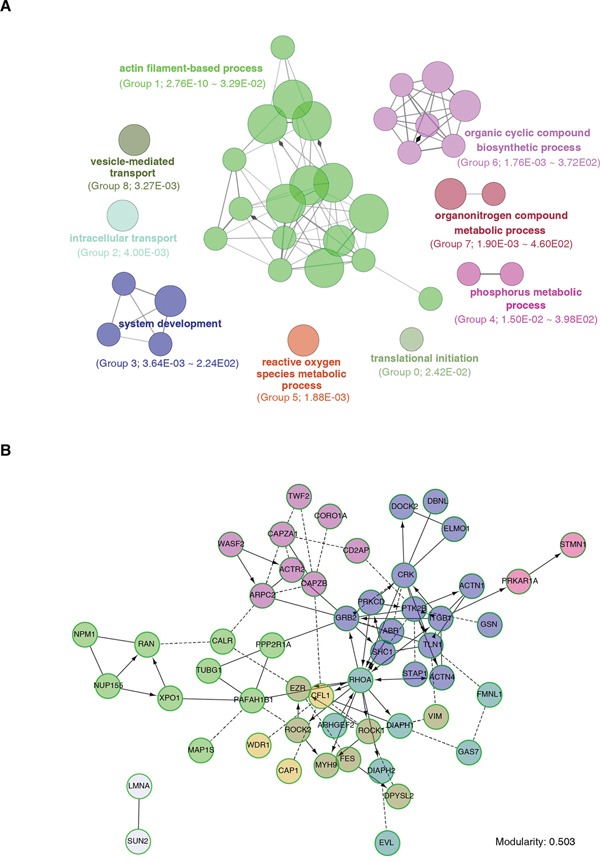
Exosomally transported proteome annotation and interaction network analysis **A.** Functional enrichment analysis with ClueGO+CluePedia. DEPs were clustered based on biological processes, and functional units were clustered into groups with various colors. The circle area depicts the gene number under such a term. The ranked group number and the range of *P* values (Fischer's Exact test) are shown between parentheses. **B.** Protein interaction network and molecular function analyses with Reactome on the Group1 genes identified by (A).

The ClueGO+CluePedia analysis enriched the 1092 significant DEPs into 36 GO terms (right-hypergeometric test with Bonferroni correction to *P* < 0.05), which were further clustered into 9 groups (Figure 3A and Supplemental Table S4). As a positive control, the vesicle-mediated transport (Group 8) was distinguished, justifying the exosomal involvement of the traced proteome (Figure 3A). Notably, 17 out of the 36 GO terms consisted of the greatest gene number were clustered into the actin filament-based process (Group 1; 76 genes) (Figure 3A). The cancer phenotype-relevant pathways included translational initiation (Group 0), phosphorus metabolic process (Group 4) and reactive oxygen species metabolic process (Group 5) (Figure 3A).

To systematically discern how the exosomally transported cytoskeleton functional unit worked together, we used ReactomeFIPlugIn from Cytoscape to cluster and analyze the Group 1 genes, taking the protein-protein interaction into primary consideration. We found that 53 out of the 76 proteins (Supplemental Table S5A) were enriched to form an interaction network with the modularity of 0.503 (Figure 3B), indicating relatively dense connection between nodes and sparse connections between modules. Molecular function analyses also showed that the major functions of these proteins were significantly focusing on actin binding, Rho GTPase binding and structural constituent of cytoskeleton (*P* < 0.001, FDR < 0.01, Supplemental Table S5B).

### CRC exosomes target at cytoskeleton rearrangement of macrophages

**Figure 4:**
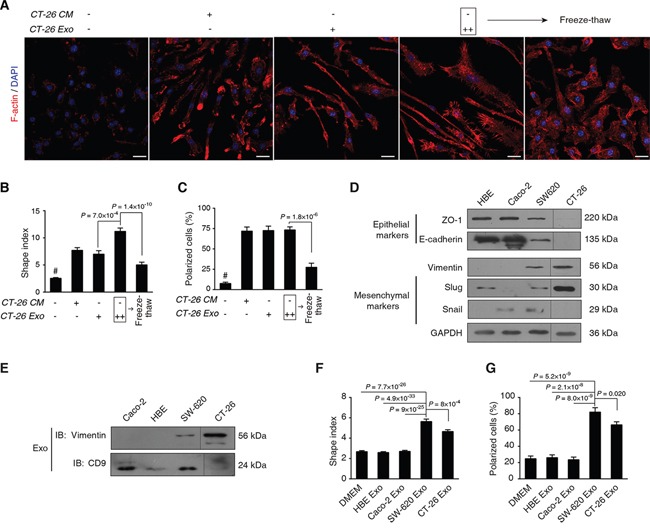
Metastatic colorectal cancer-derived exosomes sufficiently mediate cytoskeleton rearrangement of macrophages **A.** Morphological observation of F-actin rearrangement. Mouse bone marrow cells were differentiated for 3 d, followed by treatment with CT-26 CM or CT-26 exosomes (CT-26 Exo) for additional 3 d. As an interference, CT26 exosomes were disrupted in the freeze-thaw group by three quick free-thaw cycles. Images shown are F-actin (Red) and nuclei (Blue) staining by confocal microscopy. Scale bar = 20 μm. **B, C.** Statistical comparison of shape indices (B) and percentages of polarized cells (C). Image n = 20. **D.** Evaluation of EMT phenotypes of different cell types. **E.** Immunoblotting on the exosomal vimentin. Exosomes of the 4 cell lines were obtained by ExoQuick. Equal amounts of total exosome proteins (10 μg per cell line) were analyzed by IB on vimentin and CD9, respectively. **F, G.** Statistical analyses on shape indices (F) and percentage of polarized cells (G) exposed to exosomes acquired from different cell lines. Image n = 20. All statistical results shown in this Figure were obtained from 3 independent experiments. Data are shown as mean ± s.e.m. Statistical difference was tested by one-way ANOVA with Bonferroni *post hoc* multiple comparisons (two-tailed).

The above analyses suggested that exosomally transported proteins were primarily promoting the cytoskeleton rearrangement, which was biologically validated in this section (Figure 4).

We found that CT-26 exosomes had similar ability to CT-26 CM in terms of mediating the elongation and F-actin polarization of macrophages, while adding more exosomes remarkably increased the protrusions (Figure 4A); however, if we disrupted the exosome by freeze-thaw, such morphological affects were largely diminished (Figure 4A). The statistics on the shape index (Figure 4B) and F-actin polarized cells (Figure 4C) were found to support such observations, showing that the CT-26 exosome was a sufficient factor to modulate macrophage F-actin cytoskeleton rearrangements.

We used additional 3 human cell lines with different epithelial-mesenchymal transition (EMT) statuses to validate such findings. We found that the epithelial-like CRC Caco-2 and lung HBE cells shared similar features with abundant E-cadherin and ZO-1 expression, as well as low/undetectable expression of vimentin, snail or slug (Figure 4D). In contrast, highly metastatic SW620 and CT-26 cells had typical EMT features with the expression of decreased E-cadherin and ZO-1, and increased vimentin, snail and slug (Figure 4D). Vimentin was detected in both SW620 and CT-26 exosomes, while it was undetectable in Caco-2 or HBE exosomes (Figure 4E). We observed that the exosome secreted by SW620 and CT-26 cells significantly enhanced the re-arrangement of F-actin cytoskeleton in macrophages in terms of shape indices (Figure 4F) and polarized cells (Figure 4G), as compared with the Caco-2 or the HBE groups. The representative images are included in Supplemental Figure S3.

### Reproducibility of exosomally transportation of functional vimentin

We next tried to biologically verify whether the exosomally transported cytoskeleton proteins were functional, with an example to justify the proof-of-concept. We chose vimentin as such an example due to the following reasons. First, vimentin is a canonical biomarker of EMT and metastasis in numerous cancer types [41]. Second, vimentin intermediate filaments (IF) cooperate with actin and microtubules for the elongation of protrusions in invasive cancer cells [42, 43], which is useful to understand the morphological changes observed in CEEMs. Finally, the exosomally transported vimentin consisted of approximately 25% in abundance of total vimentin in CEEMs (Figure 3B, Supplemental Table S3).

**Figure 5:**
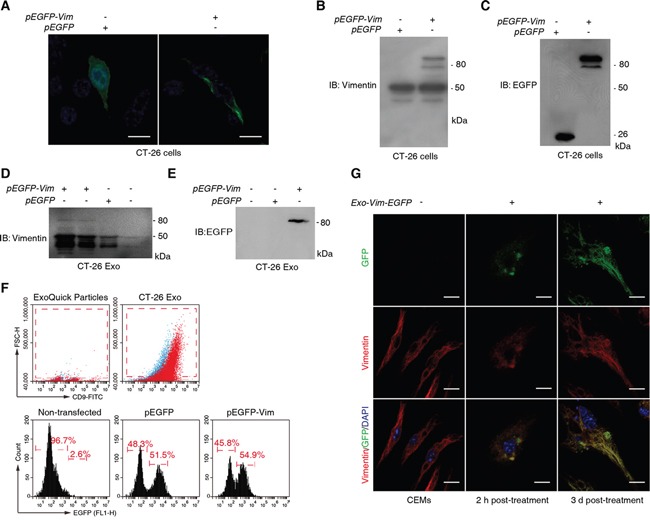
Tracing the transportation of functional vimentin from CT-26 cells to macrophages *via* exosomes **A.** Observation of intracellular expression of vimentin-EGFP fusion protein in CT2-26 cells. Cells were transfected with either pEGFP or pEGFP-*Vim* and cultured for additional 24 h prior to confocal microscopy. Scale bar = 10 μm. **B, C.** Immunoblotting validation of vimentin-EGFP expression in CT-26 cells by using anti-vimentin (B) and anti-GFP Abs (C). **D, E.** Confirmation of vimentin-EGFP incorporation in CT-26 cell-derived exosomes with anti-vimentin (D) and anti-GFP Abs (E). **F.** FCM analysis on vimentin-EGFP containing CT-26 exosomes. Exosomes were captured by ExoQuick particles and stained with CD9-FITC for FCM. Both ExoQuick particles and isotype controls (blue dots) were used to determine gating strategy on exosomes (upper two diagrams). **G.** CT-26 exosome-derived vimentin-EGFP incorporates into the intermediate filament of macrophages. BMMs were exposed to the CT-26 vimentin-EGFP containing CT-26 exosomes and harvested at 2 h and 3 d post-treatment, followed by confocal microscopic observations. Scale bar = 10 μm.

We then endogenously expressed the fusion protein of vimentin-EGFP (VIM-EGFP) in CT-26 cells, and visualized its incorporation in the vimentin IFs (Figure 5A). The intracellular expression of VIM-EGFP was confirmed by the immunoblotting (IB) assays with anti-vimentin (Figure 5B) and anti-GFP (Figure 5C) Abs, respectively. We found that Vim-EGFP could be encapsulated in the CT-26 exosome as detected by the IB with anti-vimentin (Figure 5D) and anti-GFP (Figure 5E) Abs. In addition, we used flow cytometry (FCM) to quantify the fraction of Vim-EGFP containing exosomes (Figure 5F). The size of the ExoQuick particle is less than 30 nm in diameter (enquiry from the manufacturer). As the forward scatter (FSC) was proportional to sphere size, we gated the dots that had greater FSC than ExoQuick particles as exosomes. In addition, we used an exosome biomarker CD9 to confirm the exosome detection (Figure 5F). By using the same gating strategy, we detected that over 54% exosomes isolated from the pEGFP-*Vim* transfected CT-26 cells were EGFP-positive (Figure 5F).

We next treated CEEMs with Vim-EGFP containing CT-26 exosomes (Figure 5G). As a negative control for EGFP fluorescence, the CEEMs prepared with non-transfected CT-26 cells were used. At 2 h post-exosome treatment, the colocalization of vimentin and GFP was observed and the incorporation of Vim-EGFP into IFs had already be recognizable (Figure 5G). With prolonged culture until Day 3 post-exosome treatment, Vim-EGFP was observed to be remarkably incorporated in the CEEM IFs, accompanied by cellular elongation (Figure 5G).

### Exosome-specific incorporation of cancer cell proteome

**Figure 6:**
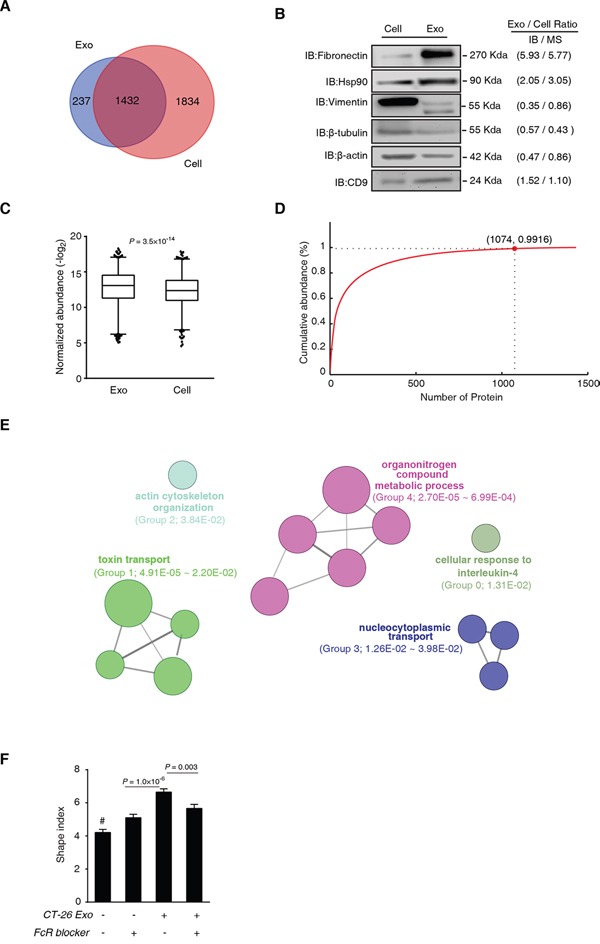
CT-26 exosome proteome is not a random incorporation of CT-26 cell proteome **A.** MS identification comparison of CT-26 cells and CT-26 exosomes. **B.** Immunoblotting validation of label-free MS quantification. The grey-scale comparison of protein expression ratio of exosome versus cell (Exo/Cell ratio) between IB and MS is shown on the right side of the IB images. **C.** Statistical comparison of the protein abundance distribution of the overlapped proteins identified in CT26 cells and CT-26 exosomes. Box-plot shows the median, quantiles and 5% outliers, respectively. KS test was used to test the significant difference in abundance distribution. **D.** Cumulative curve of CT-26 exosome proteins. Horizontal axis indicates proteins from the maximum to the minimum abundance in a ranking order. The red dot depicts the first three quartiles, and the cumulative protein abundance at this point. **E.** ClueGO+CluePedia analyses of the exosomally enriched proteins in the first 3 quartiles in (D). **F.** Effects of FcR blocking and CT-26 exosomes induced macrophage cytoskeleton rearrangement. Day 3 BMMs were treated by CT-26 exosomes with or without FcR-blocker (10 μg/mL) for additional 3 d. ^#^*P*<0.0001, compared with any of the other groups. All the images were acquired from five random high power fields of the coverslips, and more than 200 cells were measured for each group. Data are shown as mean ± s.e.m. Statistical difference was tested by one-way ANOVA with Bonferroni *post hoc* multiple comparisons (two-tailed).

We then asked whether the CT-26 exosome proteome was a random subset of CT-26 cell proteome. With the criteria of unique peptide count ≥ 2 and FDR control, we identified 1669 and 3266 confident proteins from CT-26 exosomes and CT-26 cells, respectively (Supplemental Table S6A&S6B), with 1432 overlapped identifications (Figure 6A). The relative abundance ratio information of these overlapped proteins can be found in Supplemental Table S6C. We randomly tested 6 proteins with different MWs by IB, and consistent trends were observed between IB and label-free MS quantifications (Figure 6B). We found that the abundance distribution of the 1432 proteins were significantly different in exosomes from those in CT-26 cells (Figure 6C).

To discern the functional units selectively secreted by the exosome, we plotted cumulative abundance curve of the 1432 exosome proteins ranked from the maximum to the minimum in abundance (Figure 6D). We found that proteins in the upper three quartiles (1074 proteins) cumulated over 99% of the total exosomal protein abundance (Figure 6D). From these 1074 proteins, we acquired 477 exosomally enriched proteins, whose relative abundance ratio of exosome to cell was greater than 1. These proteins were enriched in 14 GO terms that were further clustered into 5 groups, including actin cytoskeleton organization (Group 2), cellular metabolic and biosynthetic associated process (Group 4) and intracellular transportation (Group 1 and Group 2) (Figure 6E and Supplemental Table S7). Thus, the exosomal incorporation of functional units, especially the cytoskeleton unit, is not a random event, but directionally secretion of cancer cells.

The specific transportation of cytoskeleton functional units implicated that there could be a mutual recognition mechanism between CT-26 exosomes and macrophages. Fc-receptor (FcR) is a known phagocytosis- and membrane fusion-relevant molecule, highly expressed in numerous immune cells, especially in macrophages. We thus posit that cytoskeleton-centric exosomes may be recognized by macrophages *via* FcR. We then used the CT-26 exosome-induced BMM cytoskeleton rearrangement model to test the FcR blocking effect (Figure 6F). We found that although the FcR blocking could cause significant increase of the BMM shape index, such blocking could significantly reduce the macrophage shape index that was significantly promoted by CT-26 exosomes (Figure 6F; Supplemental Figure S4).

## DISCUSSION

We report here for the first time that the CRC cell-secreted exosome is a sufficient factor to transform cancer-favorable macrophages, in which cytoskeleton-centric proteome serves as one of the top functional units. By reaching the homing sites, such a mechanism represents a survival strategy of circulating CRC cells to swiftly amplify the *in situ* cancer-associated inflammation *via* existing and newly educated TAMs.

We demonstrated a feasibly new strategy to define the endpoint and exosomally transported proteome in target cells. This allowed us to identify and relatively quantify those proteins that were physically enter or attach to the target cells *via* exosomal transportation. This strategy took the advantage of the optimal labeling efficiency of SILAC [44, 45], and the deep proteome coverage of current shotgun MS analyzers [46, 47].

Although cytoskeleton proteins are readily detectable in exosomes from numerous cell types [28, 48], the role of these exosomal cytoskeleton units in the tumor microenvironment was largely unknown. The abundant transportation of cytoskeleton-centric proteins was not initially expected. For example, vimentin was found to take over ∼25% of its respective total proteins in the target macrophage. Here, we showed that being encapsulated in and delivered by exosomes were necessary for the functionality of these proteins. Indeed, cytoskeleton rearrangement is a primary phenotype of monocyte/macrophage activation and maturation [49, 50]. In agreement, we found that cancer exosomes promoted cancer-associated inflammation in terms of cathepsin B activity, pro-inflammatory cytokine secretion and proportion of polarized cells in CEEMs.

We statistically and experimentally showed that these cytoskeleton protein cargos of exosomes were not a random subset of cellular proteome, implicating the purposed secretion. Favorable to this rationale, we previously defined a specific preference of translation initiation on genes that regulate cellular movement and cytoskeleton, with over 4-fold greater translation ratios in cancer cells than those in normal epithelial cells [51–53]. Furthermore, active secretion behavior has been recognized as an important feature of CRC cells [54, 55]. These knowledges can be connected to picture the CRC cell behavior as: intensified protein production, purposed exosomal incorporation, active secretion, and functional endpoint transportation in macrophages.

Interestingly, we found that the exosomal education on macrophages were linked to the EMT status of metastatic CRC cells. Indeed, there are numerous comparable findings in the field regarding hypoxia, EMT and exosomes. As an example, hypoxia has been found to mediate the EMT of glioma cells, while the exosome serves as a critical promoter in this process [13]. A recent study has revealed that the proteome and mRNA composition profile of the exosome reflects the hypoxic status of glioma cells [56]. Comparably, EMT is also a determinant for CRC progression that can be regulated by the RhoA and Rac-1 signaling pathways [57].

Furthermore, FcR-mediated mutual recognition of CRC exosomes and macrophages potentially explains the amplified cytoskeleton units in the exosomally transported proteome identified in macrophages. By blocking FcR, we at least partially inhibited the exosome-induced F-actin cytoskeleton rearrangements. Favorably, Lesourne *et al* have reported the binding of FcR with cytoskeleton proteins, such as F-actin, filamin-1 and SHIP1 [58].

In conclusion, we revealed an active and necessary role of CRC cell-secreted exosomes in transforming cancer-favorable macrophages *via* endpoint transporting functional proteome, with cytoskeleton-centric proteins as the top functional units. This emphasizes an effort and survival strategy of cancer cells to produce excessive proteins encapsulated in exosomes to directionally and swiftly educate host cells such as macrophages to form an adaptive microenvironment at the homing site.

## MATERIALS AND METHODS

### Cell lines and animals

All animal care and experimental procedures were performed with the approval of the Institutional Animal Care & Use Committee (IACUC) of Jinan University. Specific pathogen free (SPF) male BALB/c mice, aged 6-8 w, were purchased from the Experimental Animal Center of Southern Medical University (Guangzhou, Guangdong, China). An undifferentiated and highly metastatic mouse colon carcinoma cell line [59], the CT-26 cell, was kindly provided by Dr. Xiaomin Lou, Beijing Institute of Genomics, Chinese Academy of Sciences. Human bronchial epithelial (HBE) cells, human CRC Caco-2 and SW620 cells were acquired from American Type Culture Collection. The NFκB luciferase reporter gene incorporated mouse macrophage RAW264.7 cell line was generously provided by Prof. Jiake Xu, School of Pathology and Laboratory Medicine, University of Western Australia [60]. CT-26 cells were maintained in complete RPMI 1640 media (Life Technologies, Carlsbad, CA, USA); SW620 cells were cultured in complete Leibovitz's L-15 media (Life Technologies), while HBE and Caco-2 cells were cultured in complete DMEM media (Life Technologies). All of the complete media were respectively supplemented with 10% FBS (Life Technologies) and 1% penicillin-streptomycin (Life Technologies).

### Preparation of bone marrow - derived macrophages

BMMs were prepared as we described previously with minor modifications [50, 61]. In brief, BALB/c mice were anesthetized and sacrificed, followed by bone marrow acquisition from the femur and focil with DMEM medium flushing. Red blood cells were then depleted by ACK lysing buffer (Life Technologies) and cells were cultured in DMEM-differentiation media, complete DMEM media with recombinant monocyte colony-stimulating factor (MCSF; 15 ng/mL, R&D systems, Shanghai, China).

### Isolation of exosomes

Exosomes were isolated by differential centrifugation as we previously described [34]. In brief, Cells were cultured to reach ∼80% confluence prior to serum-free medium switch for exosome secretion. Serial centrifugation at 300×*g*, 10 min and 16,500×*g*, 20 min was used to remove debris. Exosomes were enriched by a 100 kDa ultrafiltration device (Merck Millpore, Shanghai, China). The exosome size was measured by the NanoSight NS300 analyzer (Malvern Instruments Ltd, Malvern, UK) as we described previously [34]. These exosomes were used for the CEEM modeling and relevant biological experiments.

To examine the EGFP- or CD9- positive exosome with FCM, the ExoQuick-TC^TM^ kit (System Biosciences, Mountain View, CA, USA) was used for exosome isolation, following the manufacturer's instructions. Briefly, supernatants were centrifuged at 3,000×*g* for 15 min to remove debris and mixed with ExoQuick reagent with a volume ratio of 5:1 (Supernatant : ExoQuick). Exosomes were then precipitated after overnight incubation prior to FCM analyses.

### Transmission electron microscopy

We performed TEM according to Mariana et al. [62]. In brief, 20 μL of purified exosomes were layered and absorbed onto a 400-mesh copper grid, and stained with 2% Phosphotungstic acid. Samples were observed by using a transmission electron microscope (model Philips TECNAI 10) at 80 kV with the direct magnification of 65000 ×.

### Flow cytometry

The FCM detection of cellular surface biomarkers was performed following our published procedures [63]. In brief, cells were subjected to CD16/CD32 blocker (10 μg/mL, Biolenged, San Diego, CA, USA) incubation for 5 min to avoid unspecific staining. The anti-mouse mAbs used for FCM on cell samples included CD11b-PE (0.7 μg/mL, eBioscience, Shanghai, China), F4/80-FITC (2.5 μg/mL, Biolenged), CD86-FITC (7.5 μg/mL, Biolenged), CD80-PE (4 μg/mL, Biolenged) and MHC-II molecule I-A^d^-FITC (7.5 μg/mL, Biolenged). The gating strategies of all FCM analyses on surface biomarkers were determined by the corresponding isotype control of each Ab. Cells were analyzed by an Accuri C6 flow cytometer (Becton, Dickinson and Company, Franklin Lakes, New Jersey, USA). For the exosome FCM analysis, an anti-mouse CD9-FITC mAb (10 μg/mL, Biolenged) and its isotype control mAb were used. FCM data analyses were assisted by the FlowJo software version 7.6 (TreeStar, Hangzhou, China).

### Cathepsin B activity assay

Cells were stained with the Magic Red Cathepsin B Detection reagent for 1.5 h at 37°C (Immunochemistry Technologies, Bloomington, MN, USA) as we previously described [64]. Theoretically, upon enzymatic cleavage with cathepsin B, the fluorophores will be released from the probing substrates. The enzymatic activity is positively relevant to the mean fluorescence intensities (MFIs) that are detectable by FCM or confocal microscopy.

### Cytometric bead array (CBA) assay

Cytokine concentrations in supernatants were analyzed by a mouse inflammation CBA Kit (BD Pharmingen), with the procedure we previously described [63].

### Luciferase reporter assay

NFκB luciferase reporter gene activation assay was performed with a Firefly Luciferase Reporter Gene Assay Kit (Beyotime, Shanghai, China). In brief, RAW264.7 cells were sufficiently lysed and centrifuged at 10,000×g for 5 min. The cell lysate (100 μL) was mixed gently with equal volume of detecting buffer and the fluorescence intensity was detected by a GloMax^®^ 20/20 Luminometer (Promega, Beijing, China), with the parameter of 10 s detection and 2 s interval.

### Shape index assay

Cells were stained with CFDA-SE (Life Technologies) [63] or rhodamine phalloidin (Life Technologies) [50, 61] as we previously described. If the macrophage was subjected to surface FcR blocking, a FcR-blocker (CD16/CD32 blocker, 10 μg/mL, Biolenged) was used. The cellular shape index (major axis/minor axis) was measured by the ImageJ software Version 1.47e [65] as we described previously [63].

### Transwell migration assay

The CT-26 cell was plated in the upper chamber of a transwell (8 μm pore size, Corning, NY, USA) at 1.4×10^5^ cells/chamber, while the fresh or CM was loaded into the lower chamber. Cells were allowed to migrate across the membrane for 10 h prior to crystal violet staining and image acquisition with a light microscope (Olympus, Tokyo, Japan). Images were converted into binary mode and measured with the ImageJ software for the staining area coverage (area ratio of staining area to the whole area of a single view). All statistical measurements on the fluorescent and optical images were based on more than five random high power fields acquired from three independent experiments.

### Endpoint tracing of exosomally transported proteome

We adopted the SILAC method to metabolically label cells by isotopic amino acids that were optimized by Matthias Mann's group [66]. In particular, CT-26 cells were cultured for at least 6 passages in SILAC DMEM media (Thermo Fisher Scientific, Shanghai, China), supplemented with 10% dialytic FBS (Life Technologies) and 1% pen/strep as we described previously with modifications [51]. The heavy isotope labeled amino acids [73 mg/L ^13^C_6_^15^N_2_- L-lysine (Lys8) and 42 mg/L ^13^C_6_^15^N_4_-L-arginine (Arg10)] (Cambridge Isotope Laboratories, Andover, MA, USA) were used. To acquire CT-26 cell-derived and heavy isotope labeled exosomes (heavy exosomes), the SILAC labeled cells were switched to serum-free DMEM media culture for exosome secretion. At 24 h post-secretion, the labeled exosomes were collected via ultra-speed centrifugation as described above. Day 6 BMMs were subsequently treated with heavy exosomes for 2 h to allow protein transportation, in which these BMMs had been pretreated with CHX (500 ng/mL) for 1 h to avoid the reuse of isotopic amino acids. Heavy exosome-exposed BMMs were harvested (300×*g* for 5 min, at 4°C), washed twice with PBS, and lysed for protein sample preparations.

### Protein digestion and peptide fractionation

Proteins were extracted from cells or exosomes by SDS lysis buffer [1% SDS, 50 mM Tris (pH 8.1), 1 mM PMSF, protease inhibitor (Roche)] with sonication, followed by the concentration determination with a BCA kit (Thermo Fisher Scientific). We then employed the filter-aided sample preparation (FASP) [67] for the in-solution protein digestion and performed high pH RP-LC separation for the peptide fractionation as we previously described with minor modifications [47]. Specifically, peptides were loaded to a C_18_ high-pH RP-LC column (5 μm, 120 Å, 4.6×250 mm, Beijing TechMate Technology CO., LTD., Beijing, China) and eluted at 800 μL/min using a gradient from 20 mM NH_4_HCO_2_ and 2% (w/v) acetonitrile (ACN) to 4 mM NH_4_HCO_2_ and 80% (w/v) ACN over 65 min (pH = 10). The eluents were collected into 10 fractions assisted by UV absorption peak observations. Peptide samples were freeze-dried prior to MS analyses.

### Mass spectrometry analysis

Each fractionated peptide sample was reconstituted with 0.1% formic acid and 2% ACN, and analyzed with an Eksigent nano-LC tandem Triple TOF 5600 MS (AB SCIEX, Framingham, CA, USA). The detailed instrumental setting could be found in our previously publications [34, 46, 47]. All of the MS raw data were deposited in the iProX database (http://www.iprox.org, accession number: IPX00029500).

### Database search with MaxQuant

The wiff MS data files were analyzed with MaxQuant (version 1.5.2.8) by searching the Andromeda search engine against the Uniprot-Swiss mouse database (2015_02 Release, 16716 entries) as described previously [47]. In brief, the searching parameters included: enzyme, trypsin; 20 ppm for MS and 0.5 Da for MS/MS; Carbamidomethyl-Cys as a fixed modification; oxidation (M), Gln->pyro-Glu (N-terminus), and acetyl (N-terminus) as variable modifications; minimal peptide length was set to 7 residues and maximum 2 missed cleavages were allowed on tryptic peptides; decoy mode was used to evaluate the false discovery rate (FDR). Known contaminations were removed. The protein identification was considered confident only if: 1) FDR was less than 0.01 at both peptide and protein levels, and 2) at least 2 unique peptides were identified. In addition, only unique peptides were used for the SILAC quantification based on the H/L ratios (quantified unique peptide count ≥ 2).

### Label-free data analysis

The wiff MS data files were converted to the MGF format and analyzed by the Progenesis QI software for proteomics 2.0 (Nonlinear Dynamics, Newcastle upon Tyne, UK) as we previously described [47]. In brief, the MGF files were searched by Mascot software (server version 2.5.1) against the same UniProt database mentioned above. The threshold of the expect values were determined by adjusting the peptide FDR to 0.01. The Mascot search results were exported as XML files and quantified with the Progenesis QI software [47].

### Proteome bioinformatics analyses

DEPs were analyzed in the Cytoscape software (version 3.2.1) environment with various plug-ins for bioinformatics functional analysis. The ClueGO v2.2.2 + CluePedia v1.1.7 plug-in was employed to discern functionally grouped gene ontology (GO), and pathway networks to identify core functional units [68, 69]. Searches were against the GO Biological Process database (14310 terms/ Pathways, 54475 available unique gene) with the evidence from All_Experimental (EXP, IDA, IPI, IGI, IEP). The *P* value of pathway enrichment was calculated based on right-hypergeometric test and corrected with Bonferroni, and *P* < 0.05 was considered confident. Pathway clustering was calculated by using Kappa concordance test with the minimal threshold 0.4. Protein-protein interaction (PPI) and network analyses were performed by using the ReactomeFIPlugIn (Version 4.2.0. beta) for Cytoscape [70, 71]. The 2014 version of functional interactions (FIs) was used.

### Confocal microscopy

Cyto-immunohistochemistry was performed as we described previously [50, 72]. In brief, 2×10^5^cells were grown on coverslips in 24-well plates. Cells were fixed with 4% formaldehyde in PBS for 10 min at room temperature, and permeabilized by 0.1% Triton X-100 in PBS for 5 min. The rabbit anti-mouse vimentin Ab (1:100; Cell Signaling Technology, Shanghai, China) and the goat anti-rabbit IgG-FITC secondary Ab (1:50; Origene, Beijing, China) were sequentially applied. Coverslips were then mounted onto glass slides with Prolong Gold Anti-fade Reagent containing DAPI (Life Technologies) and observed with a Zeiss LSM710 confocal microscope.

### Molecular cloning and cell transfection

We cloned the mouse *Vim* gene that encoded vimentin with similar methods as described previously [72]. Total RNA was extracted from CT-26 cells by using TRIzol^®^ RNA extraction reagent (Life Technologies), following the manufacturer's instructions. The *Vim* gene was amplified by reverse-transcription PCR and validated by Sanger sequencing. The primers were: Forward: 5′- ACGTGCTAGCATGTCTACCAGGTCTGTGTCC-3′ and Reverse: 5′- ACGTAAGCTTTTCAAGGTCATCGTGATGCTG-3′. The *Vim* gene was then cloned into the pEGFP-N1 plasmid (Life Technologies) to obtain the pEGFP-VIM plasmid. To perform cell transfection, CT-26 cells were seeded in six-well plates to reach 80% confluences, followed by the addition of plasmid DNA (2.5 μg/well) and Lipofectamine LTX reagents (8 μL/well; Life Technologies).

### Immunoblotting

The IB procedure was identical to our previous reports [46, 47, 72]. The primary Abs included mouse anti- rabbit CD63 pAb (1:500; Abcam, Shanghai, China), rabbit anti- rabbit CD9 mAb (1:1000; Epitomics, Abcam), rabbit anti- rabbit HSP90 mAb (1:1000; Proteintech, Wuhan, Hubei, China), rabbit anti-fibronectin mAb, rabbit anti-vimentin mAb (1:1000; CST), rabbit anti-GFP mAb (1:1000; Epitomics), rabbit anti-E-Cadherin mAb (1:1000; CST), rabbit anti-ZO-1 mAb (1:1000; CST), rabbit anti-Snail mAb (1:1000; CST), rabbit anti-Slug mAb (1:1000; CST), rabbit anti-GAPDH pAb (1:3000; Bioworld, Nanjing, China). The secondary Ab was HRP-conjugated goat anti-rabbit Ab (1:2000; Proteintech).

### Statistics

PLGEM [39, 40], a Bioconductor package run in the *R* software environment (version 3.2.1), was used to determine data distribution, statistical power and significantly up- and down- regulated DEPs (*P* < 0.05). The abundance distribution difference in the label-free MS analyses was evaluated by Kolmogorov-Smirnov test (*KS*-test). Other measures were statistically tested by either two-tailed Student's *t*-test or one-way ANOVA with Bonferroni *post hoc* multiple comparisons based on data acquired from at least 3 independently biological replicates, assisted by the GraphPad Prism software version 6.0 (GraphPad Inc., San Diego, CA, USA); *P* < 0.05 was accepted as significant difference.

## SUPPLEMENTARY MATERIAL FIGURES AND TABLES
















