# Supplementary material for: Cytoskeleton-centric protein transportation by exosomes transforms tumor-favorable macrophages
Source: Oncotarget. 2016 Sep 1;7(41):67387–402. doi: 10.18632/oncotarget.11794 (PMC5341883; doi:10.18632/oncotarget.11794)
Supplement: Supplementary file 1 [file oncotarget-07-67387-s001.pdf]

Cytoskeleton-centric protein transportation by exosomes transforms tumor-favorable macrophages

SUPPLEMENTARY FIGURES AND TABLES

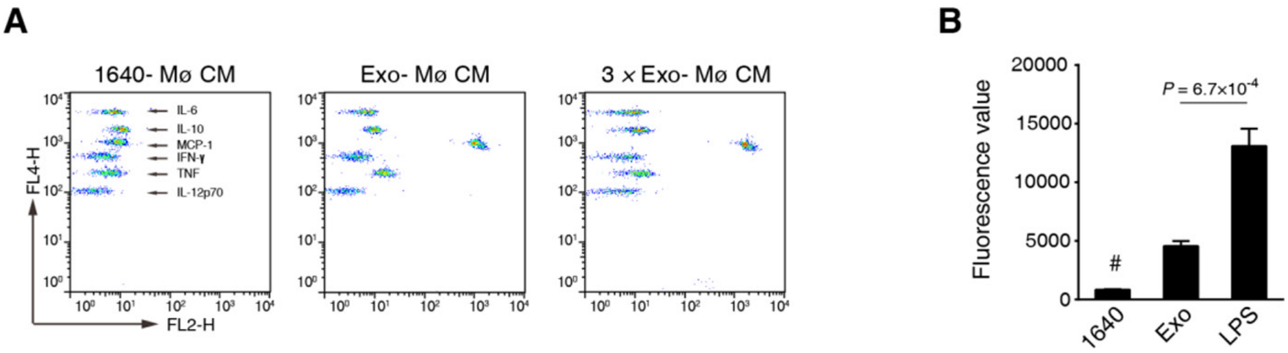

Supplementary Figure S1: A. Inflammatory phenotype analysis of CT-26 exosome-educated macrophages. (A) Representative CBA FCM results. **B.** Luciferase reporter gene assay of NFκB in the CT-26 exosome- treated RAW264.7 cells. The LPS-treated group served as a positive control for the NFκB activation. $^{\#}P<0.001$, compared with any of the other groups (n=3, two-tailed Student's *t*-test).

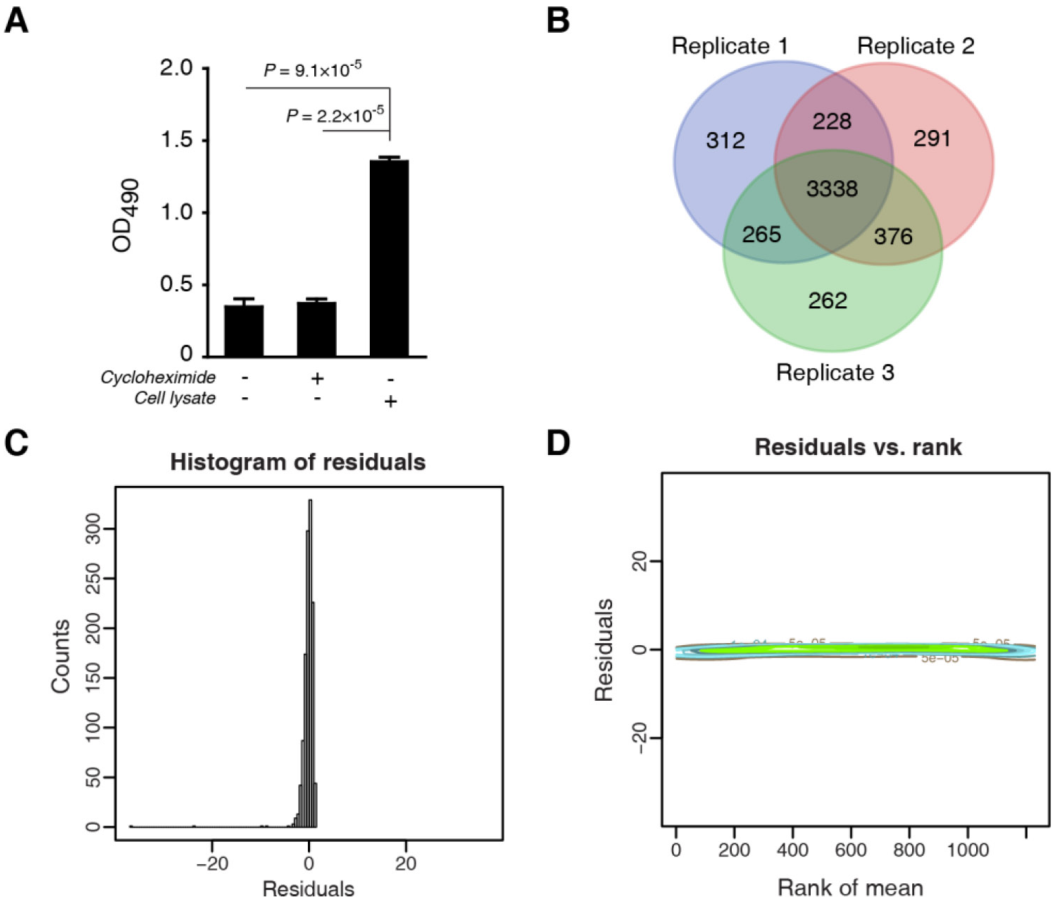

Supplementary Figure S2: Cytotoxicity assay and proteome analysis of transported proteome. A. Supernatant lactate dehydrogenase activity assay on the cycloheximide-treated BMMs. Two-tailed Student's *t*-test, n=3. **B.** Venn diagram of the protein identifications in the three CEEM MS analyses. **C, D.** Residual distribution analysis of the endpoint transported proteins in CEEMs with PLGEM.

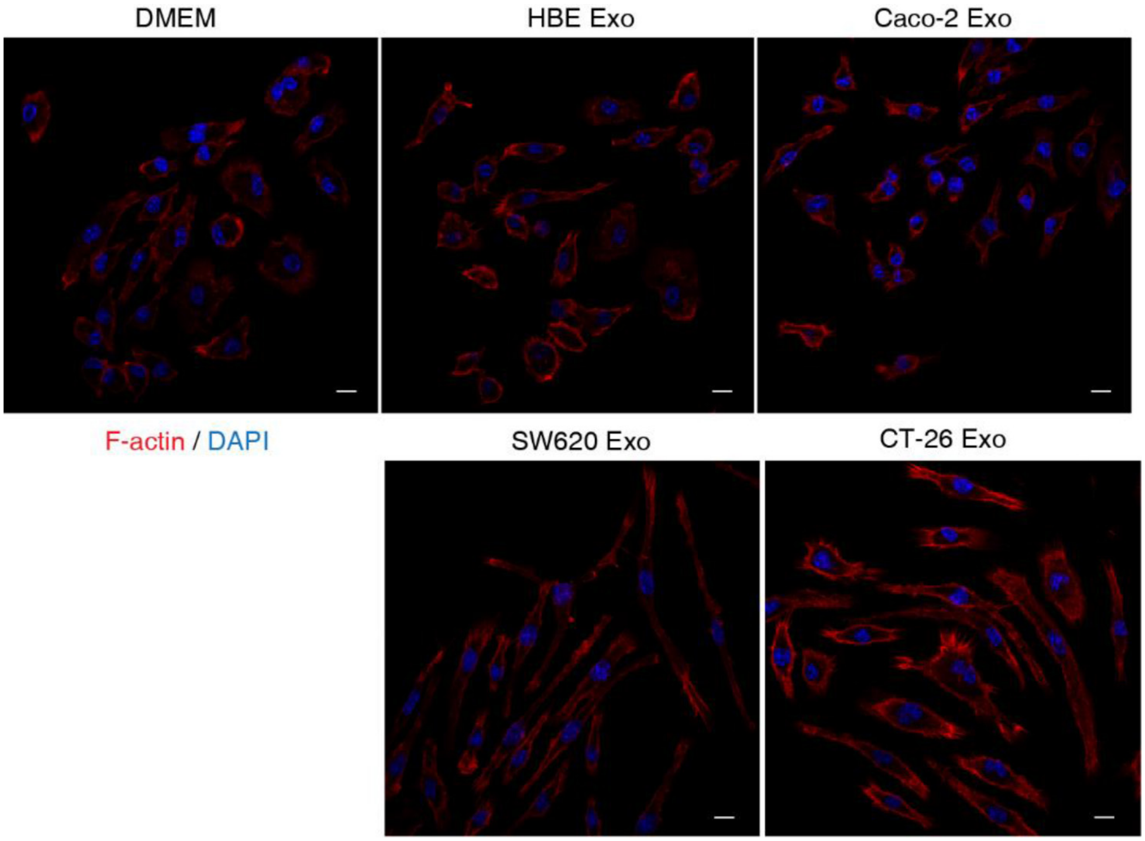

Supplementary Figure S3: Representative images of macrophages treated by exosomes isolated from cell lines with different EMT statuses. Images shown are F-actin (Red) and the nuclei (Blue) observed by confocal microscopy. Scale bar = 10 µm.

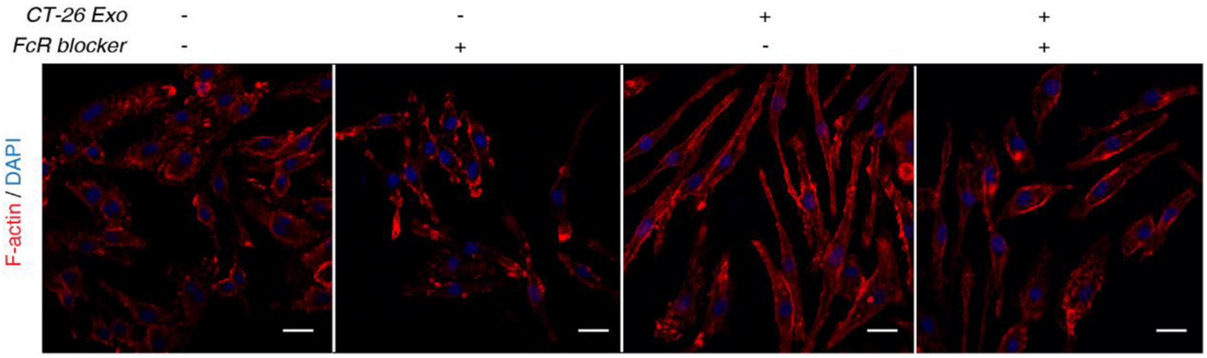

Supplementary Figure S4: Representative images of macrophages treated by exosomes and/or FcR-blocker. Images shown are F-actin (Red) and nuclei (Blue) staining by confocal microscopy. Scale bar = 10 µm.

Supplementary Table S1: Overall MS identifications of the endpoint exosomally transported proteome in CEEMs

See Supplementary File 1

Supplementary Table S2: Endpoint exosomally transported proteins repeatedly identified across all of the three replicates

See Supplementary File 2

Supplementary Table S3: Endpoint exosomally transported proteins with significant reproducibility determined by PLGEM

See Supplementary File 3

Supplementary Table S4: Gene ontology terms of the exosomally transported proteome in CEEMs

See Supplementary File 4

Supplementary Table S5: (A, B) Gene ontology terms analyzed on Group 1 genes in Supplemental Table. S4. The GO biological process (A) and the molecular function analysis by reactome (B) are shown

See Supplementary File 5

Supplementary Table S6: (A, B) Label-free MS identifications of CT26 cells (A) and CT-26 exosomes (B). (C) Comparison of relative protein abundances between CT26 cells and CT-26 exosomes

See Supplementary File 6

Supplementary Table S7: Gene ontology analysis of biological process with the exosomally enriched protein

See Supplementary File 7
